# The involvement of the wnt signaling pathway and TCF7L2 in diabetes mellitus: The current understanding, dispute, and perspective

**DOI:** 10.1186/2045-3701-2-28

**Published:** 2012-08-14

**Authors:** Wilfred Ip, Yu-ting Alex Chiang, Tianru Jin

**Affiliations:** 1 Institute of Medical Science, University of Toronto, Toronto, Canada; 2 Department of Physiology, University of Toronto, Toronto, Canada; 3 Toronto General Research Institute, University Health Network, 101 College Street, Toronto, ON, M5G 1L7, Canada

**Keywords:** Wnt, β-catenin, β-cat/TCF, FOXO, Stress, Insulin, TCF7L2

## Abstract

The Wnt signaling pathway was initially discovered for its role in tumorigenesis and the development of *Drosophila* and other eukaryotic organisms. The key effector of this pathway, the bipartite transcription factor β-cat/TCF, is formed by free β-catenin (β-cat) and a TCF protein, including TCF7L2. Extensive recent investigations have highlighted the role of the Wnt signaling pathway in metabolic homeostasis and its implication in diabetes and other metabolic diseases. Genome-wide association studies have shown that several key components of the Wnt signaling pathway are implicated in metabolic homeostasis and the development of type 2 diabetes (T2D). Despite controversial observations regarding the role of Wnt signaling in the development and function of pancreatic islets, the discovery of the association between certain single nucleotide polymorphisms of TCF7L2 and T2D susceptibility has fueled great efforts to explore the role of Wnt signaling in the function of pancreatic β-cells and glucose homeostasis. Here we have introduced our basic understanding of the canonical Wnt signaling pathway, summarized our current knowledge on its implication in metabolic homeostasis and T2D, discussed the work on TCF7L2 as a T2D susceptibility gene, and presented the controversial role of Wnt signaling and TCF7L2 in pancreatic islets as well as their potential metabolic function in other organs. We then expanded our view into the crosstalk among Wnt, insulin and FOXO signaling cascades, which further illustrates the complexity of the Wnt signaling pathway in metabolic homeostasis. Finally, we have presented our perspectives.

## Overview

The Wnt signaling pathway was initially recognized in breast and colon cancer research as well as in embryonic developmental studies of *Drosophila, Xenopus* and other organisms [1-3]. This pathway involves not only a large battery of Wnt ligands, receptors and co-receptors, but also a number of proteins that can regulate the production of the Wnt ligands, the interactions between different types of Wnt ligands and receptors on the target cells, the physiological responses of target cells to Wnt ligand binding, as well as the formation of active effector molecules. Hyper-activation of the Wnt signaling pathway, such as *via* the attenuation of the repressive machinery or the expression of a constitutively active effector, may lead to the development of colorectal and other types of tumors [4]. During the last decade, we have learned that Wnt signaling not only interacts with several other important signaling pathways in orchestrating organogenesis, but is also involved in regulating hormone gene expression and metabolic homeostasis [5,6]. Abnormalities in the Wnt signaling pathway may lead to the development of diseases other than tumors, including type 2 diabetes (T2D) and other disorders [7-11].

The major effector of the canonical Wnt signaling pathway (defined as Wnt pathway hereafter) is known as β-cat/TCF. This bipartite transcription factor is formed by free β-catenin (β-cat) and a member of the TCF protein family, including TCF7L2, which was previously known as TCF-4 [12]. In 2006, a large scale genome-wide association study (GWAS) revealed that certain single nucleotide polymorphisms (SNPs) in TCF7L2 are strongly associated with the susceptibility of T2D [13]. This important finding was subsequently replicated numerous times globally in different ethnic groups in the last few years [14-23]. Although we are still unable to determine mechanistically how these SNPs located within intronic regions of TCF7L2 affect the risk of T2D, this association, along with the recognition of the role of the Wnt signaling in the production and function of incretin hormones and blood glucose homeostasis, has prompted us to further investigate the function of the Wnt signaling pathway in the patho-physiology of T2D and other metabolic disorders [24].

Genetic variations of several other components of the Wnt signaling pathway were also shown to be involved in the susceptibility of diabetes or glucose homeostasis. In addition, Wnt signaling pathway deficiency was found to cause osteoporosis [25,26]. This is due to the existence of crosstalk between the developmental Wnt signaling pathway and the aging related FOXO/stress signaling pathway [26,27]. In this review, we have introduced the basic concept of the Wnt signaling pathway, summarized the current studies on the role of the Wnt signaling pathway in metabolic homeostasis and the development of metabolic diseases, presented current disputes on the function of Wnt signaling in pancreatic islet β-cells, and discussed the extensive recent work on TCF7L2 as a diabetes susceptibility gene. We have then further expanded our view into the crosstalk among the Wnt, insulin and FOXO signaling cascades, which allowed us to appreciate the complexity of the Wnt signaling pathway and to present our perspectives.

## Introduction of the wnt signaling pathway

In 1982, Nusse and Varmus discovered the first Wnt ligand-encoding gene, Int-1, in their breast cancer research [3]. This proto-oncogene was later renamed Wnt-1, since it shares strong amino acid sequence homology with the *Drosophila* Wingless (wg), which is important for segment polarity of the insect [28]. Today, there are 19 Wnt genes identified in rodents and humans. Wnt ligands are secreted glycoproteins which mainly exert their functions *via* the selective interaction with more than a dozen seven-transmembrane domain Frizzled receptors as well as the co-receptor known as low-density lipoprotein receptor-related protein 5 or 6 (LRP5/6).

**Figure 1:**
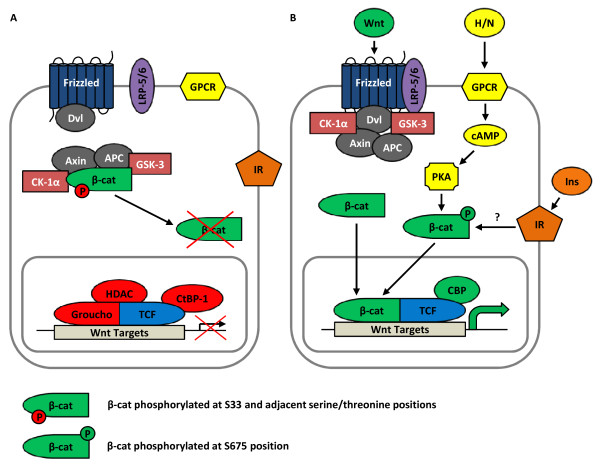
**Schematic of the canonical Wnt signaling pathway. A)** In the absence of Wnt ligands, β-cat is sequestered in the cytoplasm by the destruction complex consisting of APC, axin, GSK-3, and CK-1α, leading to its degradation by the proteasome. TCF and Groucho bind to the promoters of Wnt target genes and repress their expression. **B)** Upon binding of a Wnt ligand to the Frizzled receptor and LRP-5/6 co-receptor, the destruction complex is disrupted by Dvl. In turn, β-cat enters the nucleus where it forms the bipartite transcription factor β-cat/TCF and stimulates Wnt target gene expression. GPCR, G-protein coupled receptor. Dvl, dishevelled. APC, adenomatous polyposis coli. GSK-3, glycogen synthase kinase-3. CK-1α, casein kinase 1α. IR, insulin receptor. HDAC, histone deacetylase. CtBP-1, C-terminal binding protein-1. H/N, hormone/neurotransmitter. PKA, protein kinase A. Ins, insulin. CBP, cAMP response element-binding (CREB) binding protein.

The key effector of the Wnt signaling pathway is the bipartite transcription factor β-cat/TCF, formed by free β-cat and a member of the TCF family [TCF-1/TCF7, LEF-1, TCF-3/TCF7L1 and TCF-4/TCF7L2]. Free β-cat concentration in the cytosol of resting cells is tightly controlled by the proteasome-mediated degradation process through the actions of adenomatous polyposis coli (APC), axin/conductin, and the serine/threonine kinases glycogen synthase kinase-3 (GSK-3) and casein kinase Iα (CK-1α, Figure 1A) [29,30]. APC and axin serve as the scaffold, while GSK-3 and CK-1α phosphorylate certain serine (Ser) residues at the N-terminus of β-cat, including the Ser33 position. Once β-cat is phosphorylated at the N-terminal positions, it is ubiquitinated and degraded by the proteasome. Following binding of a Wnt ligand to the Frizzled receptor and LRP5/6 co-receptor, an association is made between the Wnt receptors and Dishevelled (Dvl), an event that triggers the disruption of the complex that contains APC, axin, GSK-3, and β-cat, thus preventing the phosphorylation-dependent degradation of β-cat. This leads to the translocation of β-cat into the nucleus, the formation of the β-cat/TCF complex, and the activation of β-cat/TCF (or Wnt) downstream target genes (Figure 1B).

GSK-3 has been recognized as an important negative modulator of the Wnt signaling pathway [31]. Lithium and other inhibitors of GSK-3 can mimic the function of Wnt ligands in stimulating the expression of Wnt downstream target genes (Figure 1B) [31]. Furthermore, the Wnt effector β-cat/TCF may also function as an effector for other signaling cascades, including insulin, insulin-like growth factor-1 (IGF-1), glucagon-like peptide-1 (GLP-1) and a number of other peptide hormones and neurotransmitters that use cAMP as a second messenger [27]. In a number of cell lineages, activation of protein kinase A (PKA) was shown to stimulate β-cat phosphorylation at Ser675, an event that is positively associated with the activation of β-cat/TCF-mediated Wnt target gene expression [32].

## Genetic studies revealing the involvement of wnt pathway components in the susceptibility of diabetes and obesity

Investigations have shown that several Wnt pathway components are important for normal lipid and glucose metabolism and hence the pathophysiology of metabolic disorders. LRP-5 and LRP-6 are co-receptors for the Wnt ligands [33,34] (Figure 1). The human LRP-5 gene is mapped within the IDDM4 region, which is linked to type 1 diabetes (T1D) on chromosome 11q13 [35-37]. A GWAS by Guo and colleagues revealed that polymorphisms of LRP-5 are strongly associated with obesity phenotypes as well [38]. The homozygous LRP-5 knockout (*LRP-5*^*−/−*^) mice showed increased plasma cholesterol levels after a high-fat diet (HFD) challenge [39]. In addition, when the *LRP-5*^*−/−*^ mice were fed with a normal diet, they also showed markedly impaired glucose tolerance [39]. Furthermore, *LRP-5*^*−/−*^ mice had a significant reduction in the levels of intracellular ATP and calcium in response to high glucose treatment, along with decreased glucose-induced insulin secretion. Finally, both Wnt-3a and Wnt-5a were able to stimulate insulin secretion in wild-type but not *LRP-5*^*−/−*^ mice [39]. Thus, Wnt-3a and Wnt-5a rely on the function of LRP-5 to facilitate insulin secretion.

Kanazawa and colleagues examined the association between genes encoding members of the Wnt family and T2D in a Japanese population [40]. They assessed 40 SNPs within 11 Wnt ligand genes, and showed that six of them exhibited a significant difference in the allele and/or genotype distributions between T2D and control subjects. Among them, one SNP within Wnt-5b was strongly associated with T2D. Wnt-5b appears to be a repressive Wnt ligand with the ability to inhibit Wnt activity and promote adipogenesis [40,41]. In addition, Wnt-5b as well as Wnt-5a are involved in coordinating chondrocyte proliferation and differentiation [42]. Thus, the GWAS to date have revealed the involvement of a Wnt ligand (Wnt-5b), Wnt co-receptor (LRP-5) and the Wnt pathway effector TCF7L2 (described further below) in the development of diabetes.

## The role of the wnt signaling pathway in adipogenesis and the function of adipocytes

The Wnt signaling pathway positively regulates bone formation [25] and negatively regulates adipogenesis [43]. Adipose tissue‒specific over-expression of Wnt-10b in mice led to ~50% lower adipose mass and the mice were resistant to HFD‒induced obesity [44]. Wnt-10b null mice, on the other hand, exhibited increased adipogenic potential [45]. The repressive Wnt ligand Wnt-5b, as mentioned above, was shown to promote adipogenesis [41].

In addition to the involvement of the Wnt signaling pathway in adipogenesis, Wnt ligands produced by adipocytes may function as paracrine or endocrine factors. Adult adipocytes express different kinds of Wnt ligands. Schinner and colleagues found that human fat-cell-conditioned medium (FCCM) stimulates the proliferation of a pancreatic β-cell line and primary mouse islet cells [46]. The FCCM was also shown to stimulate insulin secretion from pancreatic β-cells, and the stimulation can be blocked by the Wnt repressor, secreted frizzled-related protein 1 (SFRP-1) [46]. However, it is not clear as to which Wnt ligands exert the stimulatory effect on insulin secretion. Mechanisms underlying the stimulation of hormone secretion are also elusive at this stage.

## Dispute on the function of wnt signaling pathway in pancreatic islets

An early study by Murtaugh *et al.* suggests that although β-cat is essential for pancreatic acinar cell development, the loss of β-cat in a transgenic mouse model did not significantly perturb islet endocrine cell mass or function [47]. Papadopoulou and Edlund utilized the Pdx1-Cre system to delete the β-cat gene in the epithelium of the pancreas and duodenum only. They found that β-cat mutant cells had a competitive disadvantage during development. Although there was a reduction in the endocrine islet numbers during the developmental stages and the mice had pancreatitis perinatally due to the disruption of the epithelial structure of acini, mice later recovered from the pancreatitis and regenerated normal pancreas and duodenal villi from the wild-type cells that escaped the β-cat deletion [48]. Heiser *et al.*, however, found that induction of the expression of a stabilized form of β-cat at different stages results in different effects. During the early stage of organogenesis, robust expression of stabilized β-cat (the S33Y mutant) provoked changes in hedgehog and FGF-10 signaling and induced the loss of expression of Pdx-1 [49], a homeobox gene that is important for the genesis of pancreatic β-cells [50]. At a later time point in pancreas development, S33Y mutant β-cat expression enhanced islet cell proliferation and increased the size of pancreas [49]. The seemingly contradictory results reported by the above three groups can be resolved if we assume that β-cat and the bipartite transcription factor β-cat/TCF exert different functions in a precise dosage-dependent manner at different developmental stages of the pancreatic islets. Later, Rulifson *et al.* examined the effect of Wnt signaling in regulating pancreatic β-cell genesis and proliferation using both *in vitro* and *in vivo* approaches [51]. They found that purified Wnt-3a stimulated β-cell proliferation in both the mouse Min-6 cell line and primary mouse pancreatic islets, possibly through the cell cycle regulators cyclin D1, cyclin D2 and CDK-4, as well as the homeobox gene Pitx2. In the three month-old bi-transgenic rat insulin promoter (RIP)-Cre and β-cat active mice, immunohistological examinations revealed increased levels of β-cat in both the cytoplasm and nuclei of pancreatic β-cells, along with a significant increase in β-cell mass [51]. Their observations collectively suggest that Wnt signaling is necessary and sufficient for islet cell proliferation [51]. However, it is still not clear how Wnt is mechanistically involved in the embryonic genesis of pancreatic β-cells.

TCF7L2 knockout mice were generated by Korinek and colleagues in 1998 [12]. TCF7L2^−/−^ mice die shortly after birth, associated with the lack of proliferative compartments in the prospective crypt regions between the intestinal villi [12]. No examination was performed on potential abnormalities in pancreatic development or metabolism in these knockout mice during the neonatal stages. Two additional TCF7L2 knockout mouse lines were generated recently by separate groups [52,53]. The knockout mouse study by Savic *et al.* indicated the potential deleterious effects of TCF7L2 on glucose metabolism [52]. Briefly, neonatal TCF7L2^−/−^ mice exhibited hypoglycemia, while TCF7L2^+/−^ mice were protected from diabetes [52].

Liu and Habener examined the role of Wnt signaling in pancreatic β-cells from a different angle. They demonstrated that both TCF7L2 and β-cat function as effectors of the incretin hormone GLP-1 in stimulating β-cell proliferation [54]. They also showed that mouse pancreatic cells exhibit detectable Wnt activity, demonstrated in the TOPGAL transgenic reporter mice [55]. Utilizing the same β-cat/TCF-responsive TOPGAL mouse model, Krutzfeldt and Stoffel, however, reported that Wnt signaling is not appreciably active in the adult pancreas [56]. They suggest that abundant expression of the repressive Wnt ligand Wnt-4 is at least partially responsible for the lack of appreciable Wnt activity in the adult pancreas [56].

Together, although the Wnt signaling pathway is important for organogenesis, clarification of its role in pancreatic islet development requires further investigation. More importantly, canonical Wnt signaling may not be strong in the adult rodent pancreas. This notion is important for the exploration of the role of the T2D risk gene TCF7L2 in mediating glucose homeostasis (see below).

## TCF7L2 polymorphisms are associated with the risk of T2D

GWAS have been making tremendous influences on studies of diabetes and other genetic diseases [57-60]. During the last decade, extensive GWAS have shown that 38 SNPs are associated with T2D and an additional two dozen SNPs are associated with glycemic traits [59]. Among them, the T2D risk SNPs located in the TCF7L2 gene are the most exciting ones.

**Figure 2:**
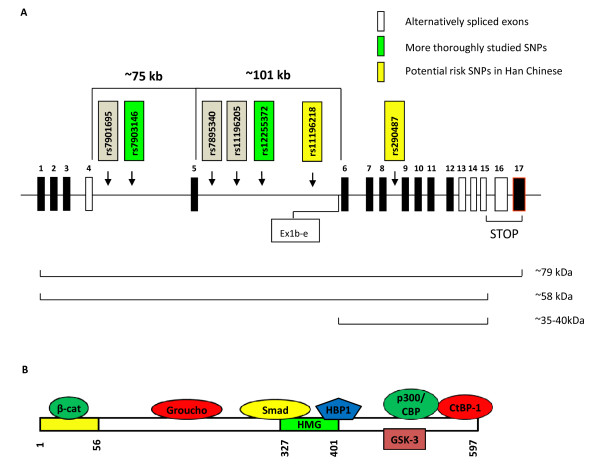
**TCF7L2 genetic structure, T2D risk SNP locations, and protein structure. A)** The human TCF7L2 gene, located on chromosome 10q25.3, consists of 17 exons (boxes). At least five exons are alternatively spliced (white boxes). Five SNPs were originally determined to be associated with T2D risk in a variety of ethnic backgrounds, all of which are located within the large intronic regions surrounding exon 5. Two additional T2D risk SNPs (yellow) were subsequently identified in Han Chinese populations. The TCF7L2 gene undergoes a significant amount of alternative splicing that produces a large number of transcripts which give rise to a number of isoforms. The major isoforms of size 79 and 58 kDa result from alternative stop codons. A novel transcription start site (called Ex1b-e) was recently identified upstream of exon 6 which leads to the production of a dominant-negative TCF7L2 isoform of size 35–40 kDa. **B)** The full-length TCF7L2 protein consists of two major domains including the β-cat binding domain at the N-terminal as well as the HMG-box for binding to DNA. In addition, TCF7L2 binds to a number of other factors, depicted in this figure. SNP, single nucleotide polymorphism. HMG, high-mobility group. HBP1, HMG-box transcription factor 1.

We have learned previously that a region on chromosome 10q is linked to T2D susceptibility [61,62]. In 2006, Grant *et al.* reported their major discovery on the linkage between polymorphisms in TCF7L2 and the risk of T2D [13]. Briefly, they revealed that certain SNPs within intronic regions of TCF7L2 show robust associations with T2D [13]. This discovery has drawn global attention and the findings have been replicated by numerous groups among different ethnic populations [14,17-19,22,23,58,60,63-76]. Figure 2A summarizes the structure of the TCF7L2 gene and five SNPs that were investigated by Grant *et al.* Among them, rs12255372 and rs7903146 are the most strongly associated with T2D, and subsequent reports determined that rs7903146 has the greatest effect in Caucasian populations. Figure 2A also indicates two other SNPs which are associated with T2D risk in Han Chinese by scientists in Taiwan and Hong Kong, respectively [64,75]. Furthermore, an alternative promoter located upstream of exon 6, named Ex1b-e and illustrated in Figure 2A, has recently been discovered to generate a native dominant-negative TCF7L2 protein in embryonic brain neurons [77]. Figure 2B depicts the protein structure of TCF7L2 which consists importantly of an N-terminal β-cat binding domain that is lacking in dominant-negative TCF7L2.

## Mechanistic exploration of the involvement of TCF7L2 in glucose disposal

**Figure 3:**
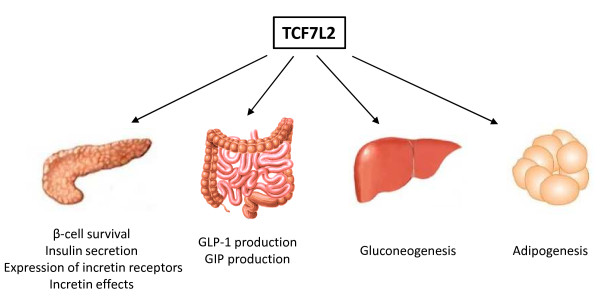
**Summary of the potential metabolic functions of TCF7L2.** The beneficial * versus * deleterious role of TCF7L2 in the pancreas is controversial and under debate. GLP-1, glucagon-like peptide-1. GIP, gastric inhibitory polypeptide.

As the TCF7L2 T2D risk SNPs are located with intronic regions, it is difficult to determine whether and how these SNPs affect TCF7L2 expression level or its alternative splicing in a given tissue. Nevertheless, great efforts have been made by numerous groups to decipher the potential role of TCF7L2 in pancreatic islets and other tissues. Figure 3 summarizes the potential metabolic functions of TCF7L2 we have learned to date.

## Controversial observations on the role of TCF7L2 in pancreatic β-cells

Initial investigations on the role of TCF7L2 in pancreatic β-cells suggested potential deleterious effects of TCF7L2. For example, Lyssenko *et al.* found that the CT/TT genotypes of SNP rs7903146 are strongly associated with the risk of T2D in two independent cohorts [23]. They observed that the pancreatic islets in T2D patients showed increased mRNA levels of TCF7L2. Furthermore, the T allele carriers exhibited a significant elevation of TCF7L2 mRNA expression in their pancreatic islets, associated with impaired insulin secretion and incretin effects [23]. As presented above, a more recent transgenic mouse study also demonstrated a potential deleterious role of TCF7L2 in mouse pancreatic islets [52].

These observations, however, are in contrast to multiple lines of work by Shu and colleagues, which revealed potential beneficial effects of TCF7L2 in pancreatic β-cells [17,18]. They found that in isolated human or mouse pancreatic islets, siRNA-mediated TCF7L2 depletion resulted in a significant increase in β-cell apoptosis and decrease in β-cell proliferation, associated with the attenuation of glucose-stimulated insulin secretion [18]. In contrast, over-expression of TCF7L2 protected islets from glucose- and cytokine-mediated apoptosis [18]. In another study, while they showed increased TCF7L2 mRNA expression levels in the islets of various rodent T2D models, they demonstrated that TCF7L2 protein levels were actually decreased [17]. In parallel, expression of TCF7L2 as well as the GLP-1 receptor (GLP-1R) and GIP receptor (GIPR) was also decreased in islets from humans with T2D as well as in isolated human islets depleted of TCF7L2 *via* siRNA treatment [17]. Finally, the stimulation of insulin secretion by glucose, GLP-1 and GIP, but not KCl or cAMP, was impaired in TCF7L2 siRNA-treated isolated human islets, while the loss of TCF7L2 resulted in decreased GLP-1 and GIP-stimulated AKT phosphorylation, and AKT-mediated FOXO phosphorylation and nuclear exclusion [17]. These findings suggest that β-cell function and survival are positively regulated by the expression of TCF7L2 in T2D. The authors note that the earlier observations showing increased TCF7L2 mRNA expression levels in T2D may actually be consistent with their findings because protein levels of TCF7L2 are oppositely regulated. Whether the expression of TCF7L2 protein is actually decreased in T2D patients or carriers of risk SNPs has not been reported.

The seemingly controversial conclusions made by different groups on the beneficial *versus* deleterious role of TCF7L2 in pancreatic β-cells could also occur due to the expression of different isoforms of TCF7L2 in a cell-type specific manner [17,18,23,52,67]. A recent study demonstrated that different isoforms of TCF7L2 in β-cells had opposite effects on β-cell survival, function, and Wnt activation [78], although no significant association has been observed between T2D risk SNPs of TCF7L2 and the alternative splicing of TCF7L2 [79]. Clearly, further investigation is warranted to clarify the role of TCF7L2 in pancreatic islets.

## The wnt signaling pathway and TCF7L2 regulate the expression and function of the incretin hormones

The role of TCF7L2 in hormone gene expression was initially demonstrated by our group [80]. We found that expression of the proglucagon gene (gcg) and production of GLP-1 were stimulated in intestinal endocrine L cells by lithium, which mimics the activation of Wnt signaling by Wnt ligands. The positive regulation of gut gcg expression by Wnt signaling was then confirmed using a constitutively active β-cat mutant and dominant-negative TCF7L2 [5,80,81], as well as the use of the Wnt ligand Wnt-3a (unpublished data of Chiang and Jin). Mechanistically, the activation is due to the binding of β-cat/TCF7L2 to the G2 enhancer element of gcg promoter, demonstrated by quantitative chromatin immunoprecipitation (ChIP) [5].

Another incretin hormone, GIP, is produced by intestinal endocrine K cells [82]. The mouse small intestinal cell line STC-1 expresses both GLP-1 and GIP. García-Martínez demonstrated that both lithium and Wnt-3 not only stimulate gcg expression, but also increase GIP transcription in this cell line [83]. Lithium or Wnt-3 was also shown to increase both cytoplasmic and nuclear β-cat contents. Interestingly, they revealed *via* ChIP that lithium treatment increased the occupancy of β-cat and LEF-1 to the GIP gene promoter, associated with reduced occupancy of TCF7L2 and the nuclear co-repressor HDAC1. They hypothesized that in the absence of Wnt signaling or lithium, GIP transcription is repressed through the occupancy of the GIP promoter by TCF7L2 and HDAC1. In response to increased β-cat, the β-cat/LEF-1 complex replaces the TCF7L2/HDAC complex, thus leading to the stimulation of GIP transcription and GIP production. Although this notion requires further examination, we can conclude that Wnt signaling and its effectors β-cat and TCF proteins are involved in the production of both GLP-1 and GIP, two known incretin hormones.

Pancreatic β-cells are the most important targets of GLP-1 and GIP. GLP-1 and its long-acting analogue exendin-4 (Ex-4) not only stimulate insulin secretion by β-cells, but also up-regulate pro-insulin gene expression, activate β-cell proliferation, and protect β-cell from stress induced apoptosis. Liu and Habener found that Ex-4 induces Wnt signaling in pancreatic β-cells [54]. They found that the expression of two known Wnt downstream targets, cyclin D1 and c-Myc, which are essentially involved in β-cell proliferation, can be stimulated by Ex-4. Furthermore, they showed that β-cat Ser675 phosphorylation is stimulated by Ex-4 treatment in the Ins-1 cell line. Finally, they demonstrated the essential role of TCF7L2 in both basal and Ex-4-stimulated β-cell proliferation utilizing both an siRNA approach and the TCF7L2 dominant-negative molecule [54]. Thus, Wnt signaling and its effectors β-cat and TCF proteins are important for both the production and function of the incretin hormone GLP-1 [5,84,85]. As presented above, Shu *et al.* established the positive relationship between the level of TCF7L2 and the levels of incretin receptors, including GLP-1R and GIPR [17]. Thus, the function of GIP is also indirectly regulated by TCF7L2, although more detailed mechanisms need to be explored.

## TCF7L2 and the wnt signaling pathway regulate hepatic gluconeogenesis

TCF7L2 is expressed in organs other than the gut and pancreatic islets, including liver, brain, muscle and fat tissues. As these organs are involved in mediating metabolic homeostasis as well, it is necessary and interesting to examine the metabolic function of TCF7L2 and Wnt signaling in those organs.

Lyssenko *et al.* found that the CT/TT genotypes of SNP rs7903146 were also associated with enhanced rates of hepatic glucose production [23]. A subsequent human study also demonstrated that this risk allele was associated with elevated hepatic glucose production, even in patients undergoing a hyperinsulinemic clamp [76].

The Wnt signaling pathway is known to be important in the development and zonation of the embryonic liver [86]. However, little effort has been made to explore the hepatic role of TCF7L2 and Wnt signaling in regulating glucose homeostasis in adulthood until very recently. Liu *et al.* found that starvation induced the expression of mRNAs that encode different Wnt isoforms in hepatocytes. They also demonstrated using loss- and gain-of-function models that β-cat is a positive regulator of hepatic glucose production [87]. Briefly, β-cat ablation improved glucose disposal and reduced the expression of the rate-limiting gluconeogenic genes, including phosphoenolpyruvate carboxykinase (PEPCK) and glucose-6-phosphatase (G6Pase). Over-expression of β-cat, however, produced reciprocal effects on hepatic gluconeogenesis [87]. Norton and colleagues, however, demonstrated that TCF7L2 silencing led to increased basal levels of hepatic glucose production in a rat hepatic cell line, associated with the over-expression of gluconeogenic genes [88]. Thus, TCF7L2 is a potential negative regulator of gluconeogenesis. Indeed, we found that the Wnt ligand Wnt-3a repressed glucose production in primary hepatocytes and cultured hepatic cell lines (unpublished data of Ip and Jin). These seemingly contradictory observations between the roles of β-cat and TCF7L2 can be resolved by considering the crosstalk among Wnt, metabolic insulin and the aging/stress FOXO signaling pathways (discussed further below).

## Potential metabolic effect of TCF7L2 in other organs

Wnt signaling negatively regulates adipogenesis [43] and positively regulates bone formation [25]. Furthermore, Wnt ligands released by adipocytes stimulate insulin secretion [46]. TCF7L2 is expressed in adipocytes and its expression can be down-regulated by insulin [89]. *In vitro* assays showed that insulin repressed TCF7L2 mRNA expression, and the repression can be attenuated by insulin resistance with the addition of free fatty acids palmitate or oleate. Insulin resistant human subjects express higher levels of TCF7L2 in subcutaneous adipose tissue [89]. Prokunina-Olsson and colleagues demonstrated that omental and subcutaneous adipose tissue express different alternatively spliced forms of TCF7L2. However, there is no association between the expression of alternatively spliced TCF7L2 isoforms and TCF7L2 T2D risk SNPs [16]. TCF7L2 is also expressed in skeletal muscle, although its role in glucose uptake or insulin signaling is currently unknown [79].

TCF7L2 is expressed in the brainstem, hypothalamus, and other areas of the brain. TCF7L2 knockout mice show abnormalities in their pituitary gland [90]. Since both incretin hormones and their receptors are expressed in brain neurons [91], and brain GLP-1 signaling controls satiety and peripheral insulin signaling [91,92], it is important to examine whether brain TCF7L2 play a role in energy and glucose homeostasis.

## Crosstalk among wnt, insulin and FOXO signaling pathways

**Figure 4:**
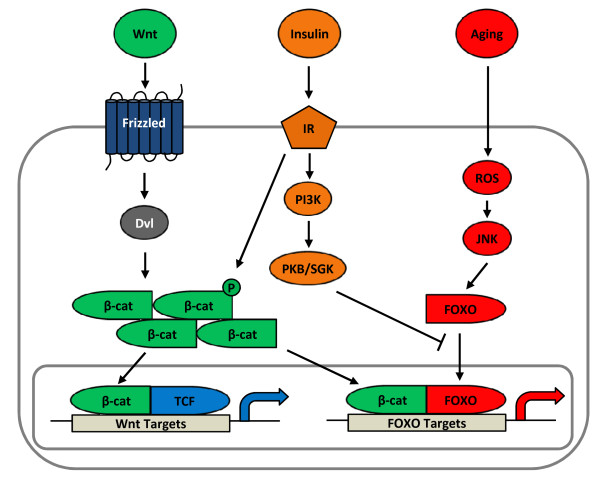
**Crosstalk among Wnt, insulin, and FOXO signaling cascades.** TCF and FOXO compete with each other for a limited pool of free β-cat. Aging and oxidative stress shift the balance in favor of FOXO signaling * via * JNK to activate downstream FOXO target genes. Contrastingly, insulin and growth factors shift the balance in favor of Wnt/TCF signaling by facilitating the nuclear exclusion of FOXO * via * PKB/SGK as well as by activating β-cat, thus resulting in the activation of downstream Wnt target genes. PKB, protein kinase B. ROS, reactive oxygen species. JNK, c-Jun N-terminal kinase. FOXO, forkhead box O. SGK, serum and glucocorticoid kinase.

FOXO proteins mediate the effects of stress and aging. The functions of FOXO proteins are negatively regulated by insulin and certain growth factors [93]. In the absence of insulin or growth factors, FOXOs are mainly located within the nuclei and up-regulate a set of target genes that promote cell cycle arrest, stress resistance, as well as apoptosis [94]. Insulin and a battery of growth factors may activate PKB or serum- and glucocorticoid-regulated protein kinase (SGK), resulting in the stimulation of FOXO phosphorylation and nuclear exclusion [93]. Essers *et al.* demonstrated an evolutionarily conserved interaction between FOXOs and β-cat, suggesting the existence of crosstalk between FOXO and Wnt signaling pathways [95]. Since FOXO can be inactivated by insulin and growth factors, scientists have explored the relationship among these three essential signaling cascades. Indeed, extensive investigations have revealed that oxidative stress plays a pathogenic role in skeletal involution, independent of aging [25,26,96-99]. More importantly, recent studies have further demonstrated a novel pathophysiological role for the interaction between FOXO proteins and β-cat in bone diseases: the reduction of β-cat/TCF-mediated gene expression. Figure 4 illustrates our current understanding of the crosstalk between the three signaling cascades. Obviously, β-cat is “not just for frizzleds anymore” [100]. The function of β-cat is bi-directional. This pivotal molecule regulates many physiological and pathological events *via* controlling cell cycle progression and cell growth. When teamed up with TCF, β-cat activates Wnt target gene expression and stimulates these two processes. When it is partnered with FOXOs, β-cat stimulates FOXO target gene expression and represses cell cycle progression and cell growth. Hence, FOXOs and TCF factors compete for a limited pool of β-cat, such that the balance is shifted towards FOXO activity during aging and oxidative stress. Insulin and growth factors, however, are able to restore balance [27].

As presented above, TCF7L2 and Wnt signaling are likely negative regulators of hepatic gluconeogenesis [88]. However, β-cat ablation improves glucose disposal and inhibit gluconeogenic gene expression [87]. How can one explain the opposite outcomes of knocking-down these two effectors of the Wnt signaling pathway on hepatic gluconeogenesis? We suggest that this involves the crosstalk among the three signaling cascades. One may speculate that free β-cat is a limiting factor for FOXOs in up-regulating gluconeogenic gene expression during fasting in response to the elevation of glucagon levels. Glucagon signaling results in the up-regulation of FOXO-mediated gene transcription to stimulate gluconeogenesis. However, upon feeding, insulin inhibits FOXO. We speculate that this will then allow free β-cat to bind to TCF7L2 and contribute to the repression of gluconeogenic gene expression. In the case of Wnt activation or TCF7L2 over-expression, FOXOs may be outcompeted by excess TCF7L2 and/or β-cat may no longer be a limiting factor. This speculation is supported by our observation that Wnt-3a represses glucose production and that insulin is able to stimulate β-cat Ser675 phosphorylation in hepatocytes (unpublished data of Ip and Jin). Alternatively, TCF7L2 may utilize a yet to be explored mechanism or co-factor to exert its repressive effect on gluconeogenic gene expression.

## Summary and perspective

Extensive investigations have shown that the Wnt signaling pathway controls hormone gene expression and mediates the function of certain hormones including GLP-1, GIP and insulin, which are critically important in glucose and energy homeostasis. We have also learned from GWAS that polymorphisms in certain Wnt ligands (such as Wnt-5b), the Wnt co-receptor LRP-5/6, as well as the Wnt effector TCF7L2, are strongly linked with T2D risk. We anticipate that further mechanistic exploration of the underlying mechanisms for these linkages will lead to the discovery of novel therapeutic targets of T2D and other metabolic disorders.

T2D is a chronic and age-dependent disorder. A fundamentally important advancement in the basic research of the Wnt signaling pathway is that β-cat/TCF mediates not only the effect of Wnt ligands, but also other cell signaling molecules during adulthood, including insulin, IGF-1, as well as peptide hormones/neurotransmitters that utilize cAMP as a second messenger [27]. These findings have not only enriched our knowledge of the function of peptide hormones/neurotransmitters, but also deepened our understanding of the existence of crosstalk among the stress/FOXO, developmental/Wnt, and metabolic/insulin signaling cascades, as presented in Figure 4. Nuclear FOXO levels rise during aging as a result of the accumulation of reactive oxygen species and JNK signaling activation. FOXOs are able to compete with TCF for a limited pool of β-cat. This leads to reduced Wnt activity, which is important for glucose and lipid metabolism. On the other hand, insulin and growth factors are able to inactivate FOXOs and hence restore the balance. The establishment of this concept creates a new perspective on the pathophysiology of T2D and other age-dependent disorders.

It is clear that TCF7L2 polymorphisms are strongly associated with the risk of T2D in different ethnic populations. Molecular mechanisms underlying this association, however, are far from understood at this time. No risk SNPs of T2D are located within the coding region of TCF7L2, or a region that can be reliably determined to have an effect on its expression or alternative splicing. In the near future, we anticipate more thorough investigations on the association between TCF7L2 SNPs and its alternative splicing in pancreatic islets and extra-pancreatic organs. Great insights can also be acquired through further exploration of the beneficial *versus* deleterious effects of TCF7L2 alternatively spliced variants in different organs.

In summary, the recognition of the involvement of Wnt components in metabolic homeostasis and the association between TCF7L2 polymorphisms and the risk of T2D have further prompted investigations into the role of Wnt signaling in the production and function of the incretin and other peptide hormones, as well as glucose disposal and insulin signaling. Although the majority of studies have been conducted in pancreatic β-cells, we anticipate further examinations on the role of Wnt signaling and its effectors in extra-pancreatic organs.

## Competing interests

The authors declare that they have no competing interests.

## Authors’ contributions

WI, YC, and TJ wrote the manuscript and approved of the final version.
